# A review protocol on research partnerships: a Coordinated Multicenter Team approach

**DOI:** 10.1186/s13643-018-0879-2

**Published:** 2018-11-30

**Authors:** Femke Hoekstra, Kelly J. Mrklas, Kathryn M. Sibley, Tram Nguyen, Mathew Vis-Dunbar, Christine J. Neilson, Leah K. Crockett, Heather L. Gainforth, Ian D. Graham

**Affiliations:** 1 School of Health and Exercise Sciences, University of British Columbia Okanagan, Kelowna, BC Canada; 2 Strategic Clinical Networks™, System Innovation and Programs, Alberta Health Services, Calgary, AB Canada; 3 Department of Community Health Sciences, Cumming School of Medicine, University of Calgary, Calgary, AB Canada; 4 Department of Community Health Sciences, Max Rady College of Medicine, University of Manitoba, Winnipeg, Manitoba Canada; 5 School of Epidemiology and Public Health, Faculty of Medicine, University of Ottawa, Ottawa, ON Canada; 6 CanChild Centre for Childhood Disability Research, Faculty of Health Sciences, McMaster University, Hamilton, ON Canada; 7 Library, University of British Columbia Okanagan, Kelowna, BC Canada; 8 University of Manitoba, Winnipeg, MB Canada; 9 George and Fay Yee Centre for Healthcare Innovation, University of Manitoba, Winnipeg, Manitoba Canada; 10 Clinical Epidemiology Program, Ottawa Hospital Research Institute, Ottawa, Ontario Canada; 11 School of Epidemiology and Public Health, University of Ottawa, Ottawa, ON Canada; 12 International Collaboration on Repair Discoveries (ICORD, University of British Columbia, Vancouver, ON Canada

**Keywords:** Knowledge synthesis, Multicenter study, Collaborative research partnerships, Integrated knowledge translation, Community-based participatory research, Stakeholder engagement, Research principles and strategies, Research outcomes and impact

## Abstract

**Background:**

Research partnership approaches, in which researchers and stakeholders work together collaboratively on a research project, are an important component of research, knowledge translation, and implementation. Despite their growing use, a comprehensive understanding of the principles, strategies, outcomes, and impacts of different types of research partnerships is lacking. Generating high-quality evidence in this area is challenging due to the breadth and diversity of relevant literature. We established a Coordinated Multicenter Team approach to identify and synthesize the partnership literature and better understand the evidence base. This review protocol outlines an innovative approach to locating, reviewing, and synthesizing the literature on research partnerships.

**Methods:**

Five reviews pertaining to research partnerships are proposed. The Coordinated Multicenter Team developed a consensus-driven conceptual framework to guide the reviews. First, a review of reviews will comparatively describe and synthesize key domains (principles, strategies, outcomes, and impacts) for different research partnership approaches, within and beyond health (e.g., integrated knowledge translation, participatory action research). After identifying commonly used search terminology, three complementary scoping reviews will describe and synthesize these domains in the health research partnership literature. Finally, an umbrella review will amalgamate and reflect on the collective findings and identify research gaps and future directions. We will develop a collaborative review methodology, comprising search strategy efficiencies, terminology standardization, and the division of screening, extraction, and synthesis to optimize feasibility and literature capture. A series of synthesis and scoping manuscripts will emerge from this Coordinated Multicenter Team approach.

**Discussion:**

Comprehensively describing and differentiating research partnership terminology and its domains will address well-documented gaps in the literature. These efforts will contribute to and improve the quality, conduct, and reporting of research partnership literature. The collaborative review methodology will help identify and establish common terms, leverage efficiencies (e.g., expertise, experience, search and protocol design, resources) and optimize research feasibility and quality. Our approach allows for enhanced scope and inclusivity of all research user groups and domains, thereby contributing uniquely to the literature. This multicenter, efficiency and quality-focused approach may serve to inspire researchers across the globe in addressing similar domain challenges, as exist in this rapidly expanding field.

**Electronic supplementary material:**

The online version of this article (10.1186/s13643-018-0879-2) contains supplementary material, which is available to authorized users.

## Background

Research partnership approaches, in which researchers and stakeholders work together collaboratively on a research project, are an important component of research, knowledge translation, and implementation [1–4]. These approaches are becoming increasingly popular, as efforts to ensconce stakeholder engagement within health care research, implementation, and improvement work converge, and are prioritized by health care systems, research funders, government, and other organizations [4]. In particular, the active integration of patients and patient-identified priorities into the research process [5–7] has become much more frequent and, in many cases, is now a mandated expectation of research teams [5, 8–10]. Research partnership approaches align well with efforts to enhance participant empowerment [11], elevate disenfranchised voices [12], and engage in real-world solution finding [13] to improve research relevance and impact [14–17].

Over the last half of a century or more, research partnership approaches have evolved within multiple research domains. A number of these approaches can be differentiated by important similarities and differences (e.g., integrated knowledge translation (IKT), participatory research, co-production, participatory action research (PAR), engaged scholarship, Mode 2 knowledge production) [14, 15, 17–19]. This variability of approach and terminology presents considerable challenges for synthesis research in the field of research partnerships, particularly in the sub-field of IKT. The dispersion and variation of relevant literature is daunting, both scientifically and logistically, and in many cases precludes attempts at more exhaustive reviews [3, 15, 20, 21]. Research partnership terminology [22–24] and definitions [15, 25] vary significantly by discipline and are still actively evolving, making IKT and other research partnership approaches difficult to capture conceptually [1–3, 21, 25–33].

IKT^1^ , in particular, has been compared and contrasted to other types of research partnership approaches. To illustrate, Salsberg and Merati [19], Salsberg [18], Jull and colleagues [15], and Bowen [34] highlight important comparisons between IKT and participatory health research: IKT and PAR, IKT and community-based participatory research (CBPR), and engaged scholarship and participatory research, respectively. However, syntheses conducted in this area to date highlight considerable limitations and challenges, such as the use of scope control techniques and the amenability of reported data for extraction and synthesis [1, 2, 21, 35–37].

In preparing this protocol, we were unable to identify a single synthesis that located, described, compared, or evaluated the literature pertaining to both IKT and related partnership research approaches within the health domain, or beyond. We identified a single review examining empirically evaluated IKT studies [21], and several syntheses focused on individual types of partnerships and relevant domains [29, 37–39]. No synthesis that comparatively described principles, strategies, outcomes, and impacts in different types of research partnership approaches was identified. The implications of these findings are significant. The co-existence of multiple, potentially relevant evidence domains is well-recognized; yet, this evidence remains largely disconnected and is often viewed superficially, or within disciplines alone. We believe that extending the comparative analysis to other domains (principles, strategies, outcomes, impacts) may help researchers more deliberately apply and rigorously evaluate research partnership approaches in future. Comparative analytics examining how and why IKT and other research partnership approaches work, the key domains (principles, strategies, outcomes, impacts), and the contextual conditions under which these approaches function may allow more deliberate and efficient stakeholder engagement [1, 21] and would represent a major step forward in the design, conduct, assessment, and impact of IKT and other research partnership approaches, in real-world settings.

This protocol describes the work plan of a newly established Coordinated Multicenter Team, focused on optimizing the quality and efficiency of IKT and other research partnership syntheses. The team has a specific interest in IKT [20, 40, 41] and applied this lens in the design of the proposed studies. Using a collaborative approach, we will build consensus strategies to address common challenges (e.g., terminology, definitions, conceptual similarities/differences, evidence volume and dispersion, logistics/resource and feasibility issues) faced by researchers attempting to synthesize the research partnership literature, including the sub-field of IKT. The three main aims of this study are to:

1. Systematically scope the literature and comparatively describe and synthesize principles, strategies, outcomes, and impacts reported in different types of research partnership approaches within and beyond health;
2. Describe and synthesize the principles, strategies, outcomes, and impacts and the accompanying research methods and tools reported in different types of health research partnership studies; and
3. Amalgamate and reflect on the collective findings and identify research gaps and future directions.

## Methods

This review protocol describes a Coordinated Multicenter Team approach to reviewing and synthesizing the key domains in different types of research partnership approaches. This work will contribute to broadening and deepening the the evidence base for research partnerships and practice.

### Coordinated Multicenter Team

A Coordinated Multicenter Team approach to plan, execute, assess, and report the proposed research syntheses will be used. The team is spread geographically and comprises clustered, multicenter teams working on complementary themes and projects. This approach will create resource and time efficiency, high productivity, and effectiveness and enhance methodological, logistical, and reporting quality within this area of the research literature. The Coordinated Multicenter Team comprises currently nine individuals (KJM, FH, KMS, TN, MVD, CJN, LKC, IDG, HG) across six academic and healthcare centers (University of Calgary, Alberta Health Services, University of British Columbia Okanagan, University of Manitoba, Ottawa Hospital Research Institute, University of Ottawa). Our work is embedded within an international integrated knowledge translation research network [42] established to systematically study and advance what is known and documented about IKT.

### Engagement of stakeholders in the proposed studies

Our Coordinated Multicenter Team will both study and employ an IKT approach in the proposed research [43]. Team members work with several stakeholder groups who have a vested interest in improving the science of IKT, including patients, in particular. A steering committee, consisting of a diverse representation of stakeholders (e.g., patients, policy and decision-makers, healthcare professionals, researchers), will be established for each individual review (see Appendix 1). Committee members will be actively involved in reviews according to their needs and preferences and according to the specific needs of each review [44–46]. At a minimum, the Coordinated Multicenter Team will engage its stakeholders in the following research phases:

- Conceptual design and formulation of the research questions
- Before starting data extraction
- Data analysis, interpretation, and dissemination of results

### Study design

**Fig. 1:**
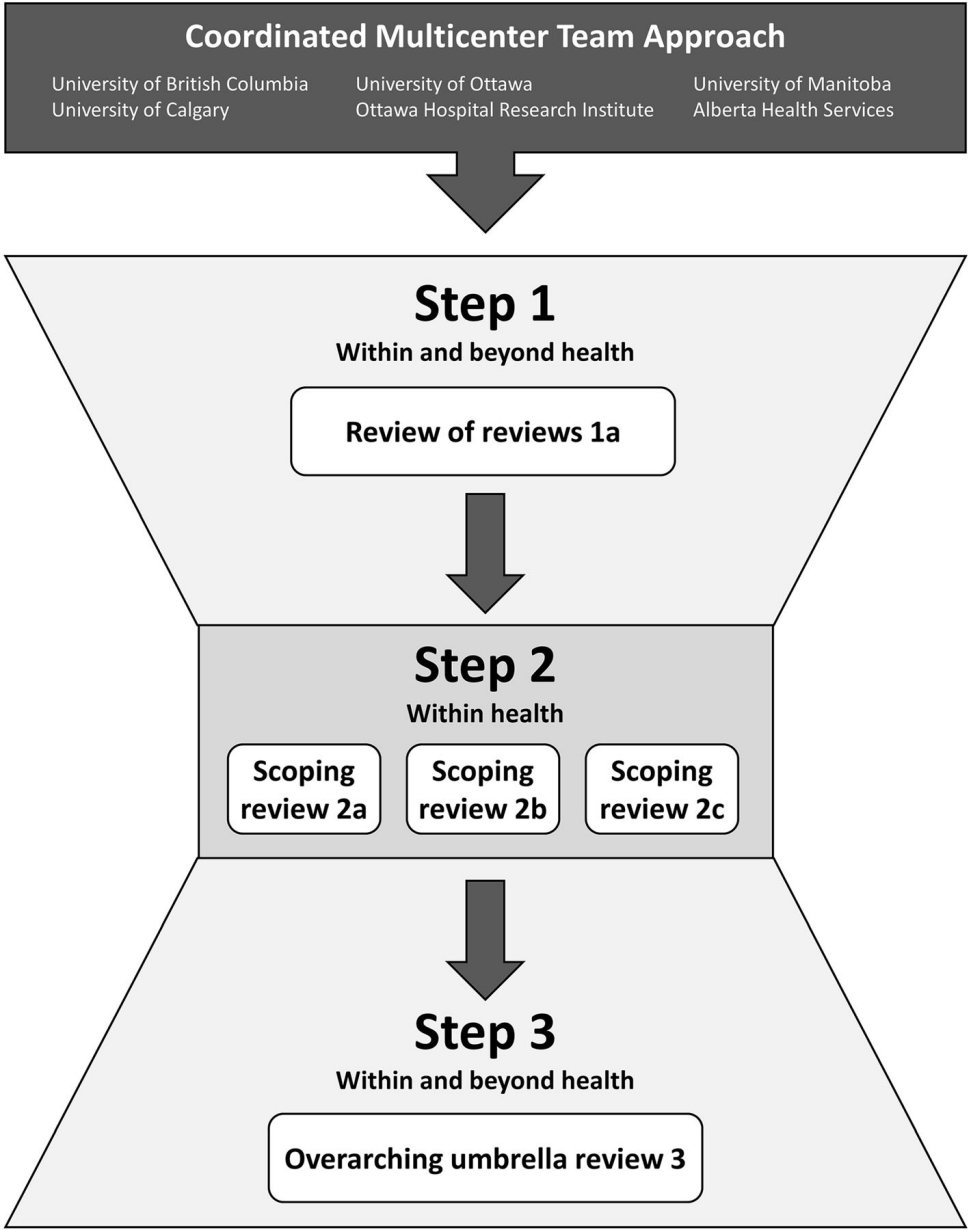
The three steps of the Coordinated Multicenter Team approach

Scoping practices outlined by Arksey, O’Malley, and other colleagues [47–51] guide our work to identify and describe the research questions, identify and select studies, abstract, collate, synthesize, and validate findings. Given the diversity of terminology and the dispersion of this literature, we will synthesize in three steps (Fig. 1). First, we will start broadly by conducting a review of reviews to comparatively describe and synthesize key domains (principles, strategies, outcomes, and impacts) for different research partnership approaches, within and beyond health (step 1). In this first step, we will identify the research partnership terminology and research scope in different practice domains in order to optimize our search strategies for subsequent steps. Secondly, using a more refined set of search strategy terms informed by the review of reviews, we will conduct several scoping reviews to describe and synthesize each key domain further in the health research partnership literature (step 2). Finally, we will amalgamate and reflect on the findings of all reviews conducted in the previous steps, using an umbrella review, to draw overarching conclusions, describe our collaborative approach and future directions, and contribute to the research agenda (step 3). A series of scoping and synthesis manuscripts will emerge a review of reviews (1a), three scoping reviews (2a–c), and one overarching umbrella review (3a).

The planning, execution, evaluation, and reporting of all reviews and findings will be guided by the Cochrane Collaboration Handbook of Systematic Reviews [52], the Preferred Reporting Items for Systematic Review and Meta-Analysis Protocols (PRISMA-P) [53], Preferred Reporting Items for Systematic Review and Meta-Analysis Equity (PRISMA-E) [54], and the emergent PRISMA-ScR for Scoping Reviews [55], and recent guidance from Pollock and colleagues for the conduct of overviews of reviews [56]. Details about the compliance with the PRISMA-P guidelines are described in Additional file 1.

### Guiding conceptual framework

**Fig. 2:**
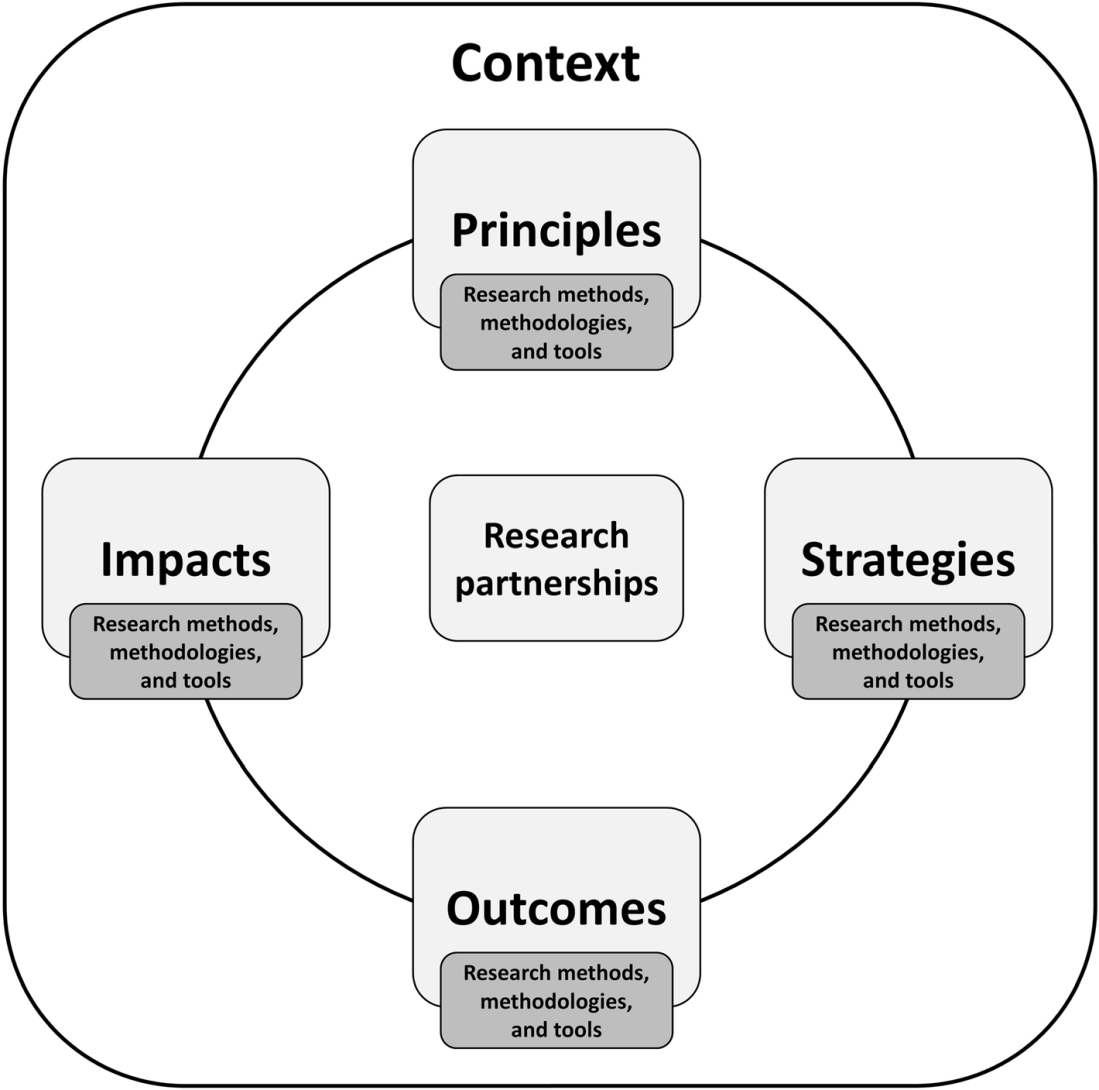
The guiding conceptual framework. All reviews will be centralized around principles, strategies, outcomes, and impacts of research partnerships. These four domains will be assessed in terms of their research methods, methodologies and/or tools

As part of protocol planning, the Coordinated Multicenter Team developed a consensus-based focused conceptual framework to guide its work (Fig. 2). Three authors (KJM, FH, HG) developed a first draft of the guiding framework based on early research questions and the PICOS for each individual review. The content of the framework was discussed with all members of the Coordinated Multicenter Team at several team meetings and revised iteratively until consensus was reached. The framework defines the topic of interest, describes key domains of research partnerships, and captures the overall intended scope and outcomes of the Coordinated Multicenter Team research agenda. This includes four key domains (*principles*, *strategies*, *outcomes*, *impacts*), and each of these domains will be assessed in terms of their *research methods*, *methodologies* and/or *tools*. Finally, both functionally and conceptually, we anticipate that the nature of each of the proposed domains will be heavily influenced by *context*.

For the purpose of this review, we will use the following operational terms and definitions:

- *Research partnerships*: “individuals, groups, or organizations engaged in collaborative research activity involving at least one researcher (e.g., individual affiliated with an academic institution) and any stakeholder actively engaged in any part of the research process (e.g., decision or policy maker, health care administrator or leader, community agency, charities, network, patients etc.)” [1, 57]. Examples of research partnership approaches include, but are not limited to, IKT, participatory research, and participatory action research.
- *Principles*: “fundamental norms, rules, or values that represent what is desirable and positive for a person, group, organization, or community and help it in determining the rightfulness or wrongfulness of its actions. Principles are more basic than policy and objectives and are meant to govern both” [58].
- *Strategies*: “observable actions designed to achieve an outcome” [59].
- *Outcomes*: “a planned, *a priori* assessment described in the study methods that is used to determine a change in status as a result of interventions, can be measured or assessed as a component of the study, and is not something of futuristic benefit”. (Adapted from the University of Waterloo Research Ethics—Definition of Outcome) [60].
- *Impact*: “identifiable benefit to, or positive influence on, the economy, society, public services, health, the environment, quality of life, or academia” [61].
- *Method*: “the techniques or procedures used to gather and analyze data related to some research question or hypothesis” [62].
- *Methodology*: “the strategy, plan of action, process, or design underlying behind the choice and use of particular methods and the choice and use of methods to the desired outcomes” [62].
- *Tools*: “an instrument (e.g., survey, measures, assessments, questionnaire, inventory, checklist, metrics, indicators, list of factors, subscales, or similar) that can be used to assess/evaluate the elements or domains of an IKT or health research partnership” [57].
- *Context*: defined as “the physical, organizational, institutional, and legislative structures that enable and constrain, and resource and realize, people and procedures” [63].
- *Facilitators*: “single or multilevel factors that are positively associated with or enhance IKT or research partnership and/or its definition, conceptualization, establishment, or conduct, design, assessment, or impact” [57]
- *Barriers*: “single or multilevel factors that are negatively associated with or hinder IKT or research partnership and/or its definition, conceptualization, establishment, or conduct, design, assessment, or impact” [57].

### Research questions

Several groups of research questions will guide our research (see Tables 1 and 2). The primary research question for the review of reviews 1a is as follows:

**Table 1:** Primary research questions and PICOS elements for the review of reviews (step 1)

	Review of reviews 1a
Framework domains	Principles, strategies, outcomes, and impacts
Research question	What differences and similarities can be identified in reported principles, strategies, outcomes, and impacts among different health and non-health research partnership approaches?
Population	Researchers, clinician scientists, trainees, policy and decision-makers, funders, patients, and other stakeholders
Intervention	Any type of research partnership approach
Comparators	Different types of research partnership approaches
Outcomes	Primary: principles, strategies, outcomes, impacts, Secondary: partnership definitions, guiding theories/models/frameworks
Study design	Any kind of literature review
Databases and time span	MEDLINE, Embase, CINAHL, PsycINFO, ERIC, Education Source, Social Services Abstracts, Sociological Abstracts, Sociology Database, Applied Social Sciences Index and Abstracts, Web of Science Core Collection, and JSTOR
Other criteria	Inclusion criteria: - Articles that describe a literature overview of research partnerships according to our definition - Articles that describe a systematic search of the literature including search terms and databases - Articles that describe a literature review on how research partnerships work (i.e., principles or strategies) *or* describe a literature review on outcomes or impacts of research partnerships - Are published in the English language. Exclusion criteria are: - Articles that do not meet our definition of research partnership - Articles that describe a review of a method or tool instead of a literature overview - Articles that describe a literature overview of assessment tools for research partnerships as the primary study aim - Articles that describe a literature review on knowledge translation and/or knowledge mobilization without a primary focus on research partnership.

*PICOS* population, intervention, comparators, outcomes, study design

**Table 2:** Research questions and PICOS elements of three scoping reviews (step 2)

	Scoping review 2a	Scoping review 2b	Scoping review 2c
Framework domains	Principles and strategies	Outcomes, impacts, and tools	Research methodologies and methods
Primary research question(s)	What principles and strategies are used to guide the different types of health research partnerships?	- What are the reported outcomes and impacts of the different types of health research partnerships? - What are the available measurement tools for assessing the outcomes and impact of the different types of health research partnerships?	What research methodologies and methods have been used to explicitly study or evaluate the partnering process underpinning health research partnership?
Population*	Researchers, clinician scientists, trainees, policy and decision-makers, funders, patients, and other stakeholders		
Intervention	Different types of health research partnerships	Different types of health research partnerships	Any study design that includes a description of the methodology and/or methods used to explicitly study or evaluate the different types of health research partnership processes
Comparators	Not Applicable	Not Applicable	No evaluation/assessment of health research partnerships
Primary outcomes	Principles and strategies	Outcomes, impacts and their characteristics, tools, tool properties	Research methodologies and methods
Secondary outcomes*	General descriptive study characteristics and partnership characteristics**		
Other criteria	Decisions regarding other inclusion and exclusion criteria, such as time span, databases, study designs, will be contingent on the terminology findings arising in Study 1a—Review of Reviews.		

*These criteria are the same in all three scoping reviews. **Examples of descriptive characteristics are author, year, research discipline, population, and context. Examples of partnership characteristics are partnership term definition, form of partnership, and partnership members. *PICOS* population, intervention, comparators, outcomes, study design

1a) What differences and similarities can be identified in reported *principles*, *strategies*, *outcomes*, and *impacts* among different health and non-health research partnership approaches?

The primary research questions for scoping reviews 2a–c are as follows:

2a) What *principles* and *strategies* are used to guide the different types of health research partnerships?

2b) What are the reported *outcomes* and *impacts* of the different types of health research partnerships, and what are the available measurement *tools* for assessing the *outcomes* and *impact*?

2c) What *research methodologies* and *methods* have been used to explicitly study or evaluate the partnering process underpinning health research partnership?

The primary research questions for the overarching umbrella review 3 are as follows:

3a) What do we currently know about *principles*, *strategies*, *outcomes*, and *impacts* in the context of research partnership approaches? What are the research gaps in the literature on research partnership approaches? What are the next steps that should be taken in the field of research partnerships?

Secondary research questions for each individual scoping review are described in Appendix 2.

### Step 1: Review of reviews

#### Search strategy

In consultation with our collaborating academic librarians (MVD, CJN), the Coordinated Multicenter Team developed a search strategy centered on capturing the following key concepts: *partnership research*, *participatory research*, *knowledge translation*, and *knowledge transfer*. We opted not to use controlled vocabularies given that preliminary inquiries confirmed poor capture of this literature by traditional health indexing. Controlled vocabulary would adversely impact precision and inflate recall [22, 23, 64].

There is great diversity in the terminologies used to express concepts associated with different types of research partnership. However, we will work on the assumption that review papers, as they synthesize existing knowledge, will use standardized terminology in their titles, abstracts, and keywords, allowing for a less complex, but still comprehensive strategy. The search will be piloted in four health databases (MEDLINE, Embase, CINAHL, and PsycINFO) to evaluate scope and feasibility. Appendix 3 describes an example of the search strategy in MEDLINE. Further refinement of this strategy will be based on our findings during an initial screening process. The refined search strategy will then be used to search a wider range of disciplines. Ultimately, the review will describe the terminology required for our subsequent reviews and allow us to identify a list of “gold standard” articles, ensuring scoping review search strategies are effective. The final search strategies for all individual databases will be available via the Open Science Framework [65, 66].

#### Electronic data sources

We will search for review papers within and beyond the health domain using the following electronic databases: MEDLINE, Embase, CINAHL, PsycINFO, ERIC, Education Source, Social Services Abstracts, Sociological Abstracts, Sociology Database, Applied Social Sciences Index and Abstracts, Web of Science Core Collection, and JSTOR.

#### Screening process and data extraction

The search will be executed by our academic librarian (MVD), and the results managed using Endnote™ X.7.5.3. De-duplication of search findings will be done according to Bramer’s method [67]. De-duplicated results will be imported into Rayyan, a web-based tool designed to facilitate the screening process of literature reviews [68]. Prior to the actual screening process, we will choose a random sample (5%) of citations to conduct calibration screening. Two members of the Coordinated Multicenter Team (FH, KJM) will take part and review the same set of citations independently. We will calculate inter-rater agreement using the kappa statistic and start the screening process once a kappa ≥ 0.6 is achieved. Where discrepancies arise, these will be discussed and resolved by consensus or failing agreement, referred to a third team member for a final decision.

The screening process will be conducted in three separate rounds. In the first round, both members will screen all citations on the title only, independently and in duplicate. Citations included by at least one team member will pass title screening and will be stored in a new database for the next screening round. In the second round, both members will screen all citations on title and abstract, independently and in duplicate, guided by the following eligibility criteria: included articles must (a) describe a literature overview of research partnerships according to our definition, (b) describe a systematic search of the literature including search terms and databases, and (c) be published in the English language. Articles that (a) do not meet our definition of research partnership and/or (b) describe a review of a method or tool instead of literature overview will be excluded and the reasons for exclusion categorized. The development of search strategies for each subsequent scoping review will be informed by the classifications formulated by this review (step 2). The third round will involve gathering full-text versions of all citations meeting eligibility criteria. Using the previously described screening calibration process, the same two team members will screen full-text review papers independently and in duplicate, discussing discrepancies to consensus or failing consensus, referring them to a third team member for a final decision. Full-text screening will then be performed based on the specific eligibility criteria aligned with our research questions (see Table 1 and Appendix 2 for more details). Once a final set of eligible review papers is generated, data extraction from full-text review papers will proceed, independently, and in duplicate, using a pre-tested data extraction tool in MS Excel. The extractable data (e.g., principles, strategies, outcomes, impacts) will be summarized for different types of research partnership approaches (e.g., IKT, CBPR, PAR). Strategies to determine the risk of bias and the methodological quality of the included review papers will be developed using published guidance [56, 69, 70].

### Step 2: Scoping reviews

#### Search strategy

Building on the findings from the review of reviews (step 1), the Coordinated Multicenter Team will refine the health research partnership search strategy. The review of reviews will be used to identify relevant terminology and definitions used in different types of health research partnership, allowing for the development of a high-quality, evidence-informed search strategy for each scoping review. The review of reviews will also supplement the creation of a “gold standard” list of articles to test subsequent searches against. This approach will require intense collaborative effort and multiple strategy alignments; we anticipate the output will generate a comprehensive, well-defined body of literature amenable to multiple reviews that are focused on specific aspects of different types of health research partnerships. The search process will be facilitated by the team’s academic librarian (MVD). For each scoping review (step 2, reviews 2a–c), three individual search strategies will be developed, to ensure reproducibility and feasibility. Search strategies will consist of two parts: (1) an overarching segment to identify different types of health research partnerships and (2) protocol-specific part(s) identifying and/or modifying the focus of each scoping review as per domains of the guiding framework (e.g., principles, strategies, outcomes, impacts). The first part of each search strategy will be the same for every scoping review (identification of research partnerships search terms), and the second part will be customized to match review-specific research questions. Strategies will be piloted in MEDLINE to determine scope and feasibility limitations and anticipate resource requirements. To optimize search quality and comprehensiveness and to refine the balance between search sensitivity and scope feasibility, draft search strategies will be scrutinized by a second academic librarian using the Peer Review of Electronic Search Strategies (PRESS) checklist [71, 72]. The team will review and consider the suggestions and make final edits to the strategy as necessary. All final search strategies for the individual databases will be available through Open Science Framework [66].

#### Electronic data sources

All scoping reviews will search for articles by using the following four electronic health databases: MEDLINE, Embase, CIHNAL, and PsycINFO. Decisions regarding additional refinements to data sources (e.g., time span, grey literature) will be specific to each scoping review and informed by the review of reviews findings.

#### Screening process

The Coordinated Multicenter Team search will be executed by an academic librarian (MVD), and results managed and de-duplicated using Endnote™ X.7.5.3, as described previously. Title and abstract screening will be performed for each scoping review separately using a pre-tested MS Excel screening tool. Screening calibration will be undertaken in two stages, by title and abstract, and then in full text, using the methods described previously (step 1), and will be carried out independently and in duplicate by two team members. Discrepancies will be discussed and resolved by screener consensus or referred to a third investigator for final resolution.

To maximize quality and ensure comparability within a very large volume of literature, we formulated general eligibility criteria for all scoping reviews for use with title and abstract level screening. We will combine these general eligibility criteria with review-specific criteria. We will include citations that involve research partnerships in the health domain. We will exclude articles that do not meet the definition of research partnership. For all excluded studies, we will track primary reasons for exclusion. After title and abstract screening, each scoping review team will proceed with the full-text screening process. Once a final set of eligible papers is generated, data extraction from full-text papers will proceed.

Screening calibration within each scoping review will be undertaken at each screening level as described previously. *A priori* agreement on common terms and definitions was achieved and will be applied during all study levels.

Aspects related to study records (risk of bias) and data (synthesis, meta-bias, confidence in cumulative evidence) will be tailored to each individual scoping review paper. Details about these aspects will be described in the individual review papers.

### Step 3: Overarching umbrella review

Finally, two researchers (HG and KS) will synthesize and aggregate findings from the review of reviews and the three scoping reviews using an umbrella review, in collaboration with review leads (FH, KJM, TN). In accordance with published guidelines for developing, conducting, and reporting umbrella reviews [73], this review will synthesize findings from the multiple reviews into one accessible and useable document. [74]

## Discussion

This review protocol outlines our Coordinated Multicenter Team approach to reviewing and synthesizing research partnerships using an innovative and collaborative review methodology. Our approach will result in a series of review manuscripts describing specific aspects of different types of research partnerships and attempt to address the documented gaps, with a specific focus and interest in IKT [20, 40, 41]. By documenting our Coordinated Multicenter Team approach, we hope to provide guidance to and inspire researchers in the same or other fields to tackle evidence bases that are challenged by scope, terminology, dispersion, and volume.

Our Coordinated Multicenter Team approach is centralized around three key aspects. First, we will *optimize research quality* by sharing knowledge and expertise among all team members. Our team currently consists of nine individuals with different backgrounds and expertise (e.g., KT, implementation, IKT, behavioral science, research partnerships, knowledge synthesis), working in six different organizations across Canada. This provides a unique opportunity to learn in collaboration and raise the quality and integrity of multiple reviews. All papers published by the Coordinated Multicenter Team will use common terms and language based on our consensus-driven and literature-based guiding framework and related terms and definitions and reporting criteria [21], unless otherwise noted (Fig. 2). The Coordinated Multicenter Team papers may be used as a template for future research to conduct studies and report on different types of research partnership (e.g., we know of at least two similarly structured systematic reviews that will cascade from this first proposed set of reviews). Our search strategies will be publicly available [65, 66], giving other researchers the opportunity to use our searches and build upon our work in refining terms and in locating and describing the nature of the evidence base for IKT and other types of research partnership approaches. In this way, we hope that our work will enhance research quality and transparency in the field of IKT and other research partnership approaches by creating a common language for reporting and planning.

Second, we will *increase capacity by maximizing synthesis team efficiency* in all stages of the review processes (e.g., search strategy development, screening process, procedural alignments for screening, extraction, dissemination). For example, we will use our findings from the review of reviews to develop an overarching research partnership-focused search strategy that will be applied in all our scoping reviews. We expect that a search strategy built upon terms and definitions comprising the breadth of the literature pertaining to research partnership will result in more focused results and will improve feasibility with well-justified search strategy controls. Syntheses can pose significant time, resource, and volume challenges [75] in fields where there is a high diversity of terminology, procedures, and literature dispersion. Our efficiency- and quality-focused collaborative review methodology offers potential strategies to overcome these challenges and may therefore contribute to the literature on review methodologies addressing efficiency and quality improvement [76–78]. Moreover, this approach allows for an enhanced scope for each review and enhances inclusivity of all research user groups and domains, thus contributing uniquely to the literature and reducing the potential for duplication of efforts.

Third, we hope *to maximize the impact* of our work by ensuring that our projects are relevant and usable to a broad audience by using an IKT approach tailored to each individual review project [43–46]. We will establish steering committees consisting of a diverse group of stakeholders for each individual review and engage them throughout the review processes (Appendix 1). Moreover, we will reflect on our own IKT approach and will share the lessons learned in the overarching umbrella review. Our Coordinated Multicenter Team approach will, therefore, meet the needs of our partners and ensure that both researchers and stakeholders can benefit from our work.

In summary, our protocol paper provides a methodological design template for future researchers to construct their own reviews or research. It contributes to the methodological refinement of review processes using multi-site collaborative teams, in which design, workflow, scientific and logistical strategy, and other efficiencies are leveraged to optimize research quality. Ultimately, we hope our efforts will contribute to and improve the quality, conduct, and reporting of the research partnership literature. Our Coordinated Multicenter Team approach may serve to inspire researchers across the globe in addressing similar domain challenges, as exist in this rapidly expanding field.

## Additional file

Additional file 1 PRISMA-P Checklist (DOCX 22 kb)

## Appendix 1—Steering committees

A steering committee, consisting of a diverse representation of stakeholders (e.g., patients, policy and decision-makers, healthcare professionals, researchers), will be established for each individual review. This appendix provides an overview of the stakeholders who will be a member of one of the steering committees.

The following partners will take part in one of the steering committees:

- Spinal Cord Injury Ontario
- Spinal Cord Injury BC
- Ontario SCI Solutions Alliance
- Northern American Spinal Cord Injury Consumer Consortium
- Miami Project
- Rick Hansen Institute
- Research Manitoba
- International Collaboration on Repair Discoveries
- Spinal Cord Injury Canada
- Michael Smith Foundation for Health Research
- Mrklas, KJ—PhD Supervisory Committee, Department of Community Health Sciences, Cumming School of Medicine, University of Calgary
- Clinicians from Hamilton Health Sciences, Toronto Rehabilitation Institute—University Health Network, Seine River Physiotherapy
- Patient research partners from the BC SUPPORT Unit, University of Calgary

## Appendix 2—Compendium of Review Protocols

*Review of reviews 1a—Comparing and synthesizing research partnership approaches*

Aim

This review of reviews aims to identify differences and similarities in principles, strategies, outcomes, and impacts reported in different health and non-health research partnership approaches in order to improve the differentiation among different research partnership approaches.

Primary research question

- What *differences and similarities* can be identified in reported principles, strategies, outcomes, and impacts among different health and non-health research partnership approaches?

Secondary research questions

- What kind of health and non-health research partnership approaches can be distinguished?
- What definitions are used for research partnerships?
- What principles, strategies, outcomes, and impacts are reported in different health and non-health research partnership approaches?
- What are the differences and similarities between research partnership within and beyond the health domain?
- What theories, models, and frameworks are used to guide review papers on health and non-health research partnership approaches?

*Scoping review 2a—Principles and strategies*

Aim

This scoping review aims to identify key *principles* and *strategies* for different health research partnerships.

Primary research question

- What *principles* and *strategies* are used to guide the different types of health research partnerships?

Secondary research questions

- How can the identified *strategies* be linked to the identified *principles*?
- What theories, models, and frameworks are used to guide the different types of health research partnerships? How do they relate to the guiding principles and strategies?
- How do the guiding principles and strategies differ between *stages in the research process* (e.g., data collection, data analysis, interpretation of findings, disseminating of findings)?
- How do the guiding principles and strategies differ between *settings*?
- How do the guiding principles and strategies differ between *populations*?
- How do the guiding principles and strategies differ between *groups of research users* (e.g., patients, practitioners, decision-makers, health organizations)?
- What *facilitators* and *barriers* are associated with the guiding principles and strategies?

*Scoping review 2b—Outcomes, impacts, and tools*

*Aim*

This scoping review aims to establish the scope, nature, and location of the global literature pertaining to the reported *outcomes* and *impacts* of different health research partnerships and *outcomes* or *impact* assessment tools.

Primary research questions

- What are the reported *outcomes* and *impacts* of the different types of health research partnerships?
- What are the available measurement tools for assessing the *outcomes* and *impacts* of the different types of health research partnerships?

Secondary research questions

- How are the different health research partnership outcomes or impact (and/or related concepts) defined and described?
- What are the key characteristics and constructs of the different health research partnership outcomes or impact assessment tools?
- What are the documented barriers, facilitators, and/or other documented influences on the development, assessment, or use of the different health research partnership outcome or impact assessment tools?
- What emergent gaps exist in health research partnership outcome or impact assessment?
- To what extent is the nature and scope of the evidence base in this area amenable to systematic review?
- What future research questions arise in the literature on health research partnership outcomes or impact assessment?

*Scoping review 2c—Research methodologies and methods*

Aim

This scoping review aims to describe the research methodologies and methods used to study or evaluate the partnering process underpinning health research partnership.

Primary research question

- What research methodologies and methods have been used to explicitly study or evaluate the partnering process underpinning health research partnership?

Secondary research questions

- How is IKT defined in studies?
- To what extent has the IKT approach been explicitly studied/evaluated in published literature?
- In what contexts has the IKT approach been explicitly studied/evaluated?

*Review 3—Overarching umbrella review*

Aim

The final paper aims to synthesize the findings from the four reviews in terms of the guiding conceptual framework.

Primary research questions

- What do we currently know about *principles*, *strategies*, *outcomes*, and *impacts* in the context of research partnership approaches? What are the research gaps in literature on research partnership approaches? What are the next steps that should be taken in the field of research partnerships?

## Appendix 3—Search strategy

The example search strategy for the review of reviews in MEDLINE.

**Table:**

Line	Search	Hits
1	(((partnership or participatory) adj2 research) or ((transfer or translation) adj knowledge) or ((“mode 2” or “mode two” or “mode II”) adj2 (knowledge or research)) or “linkage and exchange”).ti,ab,kw,kf.	4715
2	(partnership? or “community-based”).ti. and (research or assessment or measurement or outcomes or tools).ab,ti.	7484
3	review.pt. or (“scoping review*1” or “systematic review*1” or “review of the literature” or “literature review*1” or “umbrella review*1” or “review of reviews” or “mapping review*1” or “realist review*1” or “rapid review*1”).ab,ti.	2,447,648
4	1 or 2	11,025
5	3 and 4	983

The search was run on January 29, 2018

## Abbreviations

CBPR: Community-based participatory research
IKT: Integrated knowledge translation
PAR: Participatory action research

## Footnotes

1 We operationalize IKT using a recent iteration of an earlier definition [79] as: *“…a way of approaching research to increase the chances that the results will be applicable to the population under study. [IKT] is a paradigm shift that focuses on engagement with end users and the context in which they work. Essentially, it is a collaborative way of conducting research that involves researchers and knowledge-users, sometimes from multiple communities (e.g. clinicians, managers, policy makers, patients, [among others]) working together as partners in the research process*.” (Graham, Tetroe & MacLean, 2014, p.11) [80].
